# The myocardial capillary network is altered in congenital diaphragmatic hernia in the fetal rabbit model

**DOI:** 10.1590/1414-431X2023e12521

**Published:** 2023-05-15

**Authors:** A.L.A. Nour, A.T. Fabro, S.S. Batah, M. Oria, J.L. Peiro, L. Sbragia

**Affiliations:** 1 Divisão de Cirurgia Pediátrica, Departamento de Cirurgia e Anatomia, Faculdade de Medicina de Ribeirão Preto, Universidade de São Paulo, Ribeirão Preto, SP, Brasil; 2 Departamento de Patologia, Faculdade de Medicina de Ribeirão Preto, Universidade de São Paulo, Ribeirão Preto, SP, Brasil; 3 University of Cincinnati Medical College, Cincinnati Fetal Care Center, Cincinnati Children's Hospital Medical Center (CCHMC), Cincinnati, OH, USA

**Keywords:** Animal model, Congenital diaphragmatic hernia, Heart, Myocardial, Capillary

## Abstract

Congenital diaphragmatic hernia (CDH) is associated with thoracic compression of the lungs and heart caused by the herniated abdominal content, leading to cardiac modifications including pressure and vascular changes. Our aim was to investigate the experimental immunoexpression of the capillary proliferation, activation, and density of Ki-67, VEGFR2, and lectin in the myocardium after surgical creation of a diaphragmatic defect. Pregnant New Zealand rabbits were operated on the 25th gestational day in order to create left-sided CDH (LCDH, n=9), right-sided CDH (RCDH, n=9), and Control (n=9), for a total of 27 fetuses in 19 pregnant rabbits. Five days after the procedure, animals were sacrificed, and histology and immunohistochemistry studies of the harvested hearts were performed. Total body weight and heart weight were not significantly different among groups (P=0.702 and 0.165, respectively). VEGFR2 expression was increased in both ventricles in the RCDH group (P<0.0001), and Ki-67 immunoexpression was increased in the left ventricle in the LCDH group compared to Control and RCDH groups (P<0.0001). In contrast, capillary density was reduced in the left ventricle in the LCDH compared to the Control and RCDH groups (P=0.002). Left and right ventricles responded differently to CDH in this model depending on the laterality of the diaphragmatic defect. This surgical model of diaphragmatic hernia was associated with different expression patterns of capillary proliferation, activation, and density in the myocardium of the ventricles of newborn rabbits.

## Introduction

Congenital diaphragmatic hernia (CDH) is a condition marked by herniation of the abdominal organs into the thoracic cavity through a diaphragmatic defect. CDH occurs at a frequency of approximately 3 in every 10,000 births (1), but with high morbidity and mortality, despite medical and surgical progress during the last decades (2).

The physiopathology of CDH has primarily been considered due to lung compression caused by herniated abdominal organs into the fetal chest, leading to significant compression of the lungs and heart. Consequent pulmonary abnormalities are of variable degree and may be explained by the dual-hit hypothesis (3). Hypoplastic lungs are immature and smaller, showing reduced bronchial tree branching, acinar hypoplasia, and alveolar septal thickening (4).

Despite the focus of pathophysiological investigation in CDH being the lungs, the heart seems to also be affected by the compression from the herniated organ. Siebert et al. (5) documented for the first-time cardiac hypoplasia, demonstrating reduced left ventricle, left atrium, and interventricular septum mass upon *post mortem* examination of 8 hearts from newborns with left-sided CDH. In addition, echocardiographic analysis of 20 newborns was performed by Schwartz et al. (6), also confirming the presence of left-sided cardiac hypoplasia, and the authors suggested that left ventricle size could be an important predictor of overall prognosis. Experimentally, Tannuri (7) demonstrated reduced total weight and reduced ventricular wall thickness of neonatal hearts after creating a surgical model of CDH in rabbits, despite an increase in interventricular septum thickness. Pelizzo et al. (8) studied small intramyocardial vessel density by analyzing the hearts of 7 neonates with CDH *post mortem* observing a significant increase in vessel density compared with Controls.

Considering the evidence for both macro- and microscopic abnormalities in the heart of fetuses and children with CDH, we aimed to evaluate the effect of CDH in the endothelial proliferation by evaluating the immunoexpression of Ki-67 (a nuclear protein used as a marker of cell division) and the endothelial activation by studying the vascular endothelial growth factor receptor 2 (VEGFR2), an essential piece of the angiogenic process and that has been shown to be reduced in the lung of CDH rabbits (9), and capillary density by lectin staining of intramyocardial vessels.

## Material and Methods

### Ethics approval

The Institutional Ethics Board Committee for Animal Research approved the experimental research (CEUA-FMRP #100/2017).

### Animals and study design

Pregnant New Zealand rabbits (term=30 days) were used for this study (n=19). Three groups were designed: fetuses with a left sided diaphragmatic defect (LCDH-n=9), fetuses with a right sided diaphragmatic defect (RCDH-n=9), and Controls (n=9). The Control group consisted of non-operated fetuses from the same mother rabbit.

### Surgical procedure

The rabbits were housed in individual cages with access to food and water *ad libitum*.

The surgical procedure for the creation of diaphragmatic defects was conducted according to Fauza et al. (10). This well-established experimental model of CDH mimics the second phase of lung insult when the liver grows large and compresses the lung (mechanical effect). The term of rabbit pregnancy is 30-31 days and day 25 of fetal lung is like the canalicular phase of lung development (11). We performed 3 or 4 CDH for each rabbit. On the 25th day of pregnancy, rabbits were sedated with intramuscular injections of 50 mg/kg of ketamine (Cristália, Brazil) and underwent laparotomy to expose the uterine horns and identify the fetuses. The fetal thoracic cavity was accessed with left or right thoracotomy, and the diaphragm was cut with micro scissors. The fetal thorax was closed with 6-0 Prolene sutures (Ethicon, USA). After closure of the uterine incision with 5-0 Prolene sutures, 2 mL of warm saline was injected into the uterine cavity to replace lost amniotic fluid. The abdominal wall was closed on a single plane with 2-0 Vycril sutures (Ethicon), and the skin was closed with a continuous 4-0 nylon suture (Ethicon). The pregnant rabbits then received 25 mg/kg of cefazolin and 2.5 mg/kg of medroxyprogesterone acetate (Pfizer, Belgium), intramuscular, for infection and preterm labor prophylaxis. After the procedure, the animals were kept with supplementary oxygen in a temperature-controlled environment for 2 h until recovery from surgery. Harvest occurred five days after the operation. Newborns were identified, weighed with an analytical scale (APX-200, Denver Instruments, USA). The mother was then sacrificed with a lethal dose of sodium thionembutal (Cristália).

### Harvest and tissue processing

**Figure 1:**
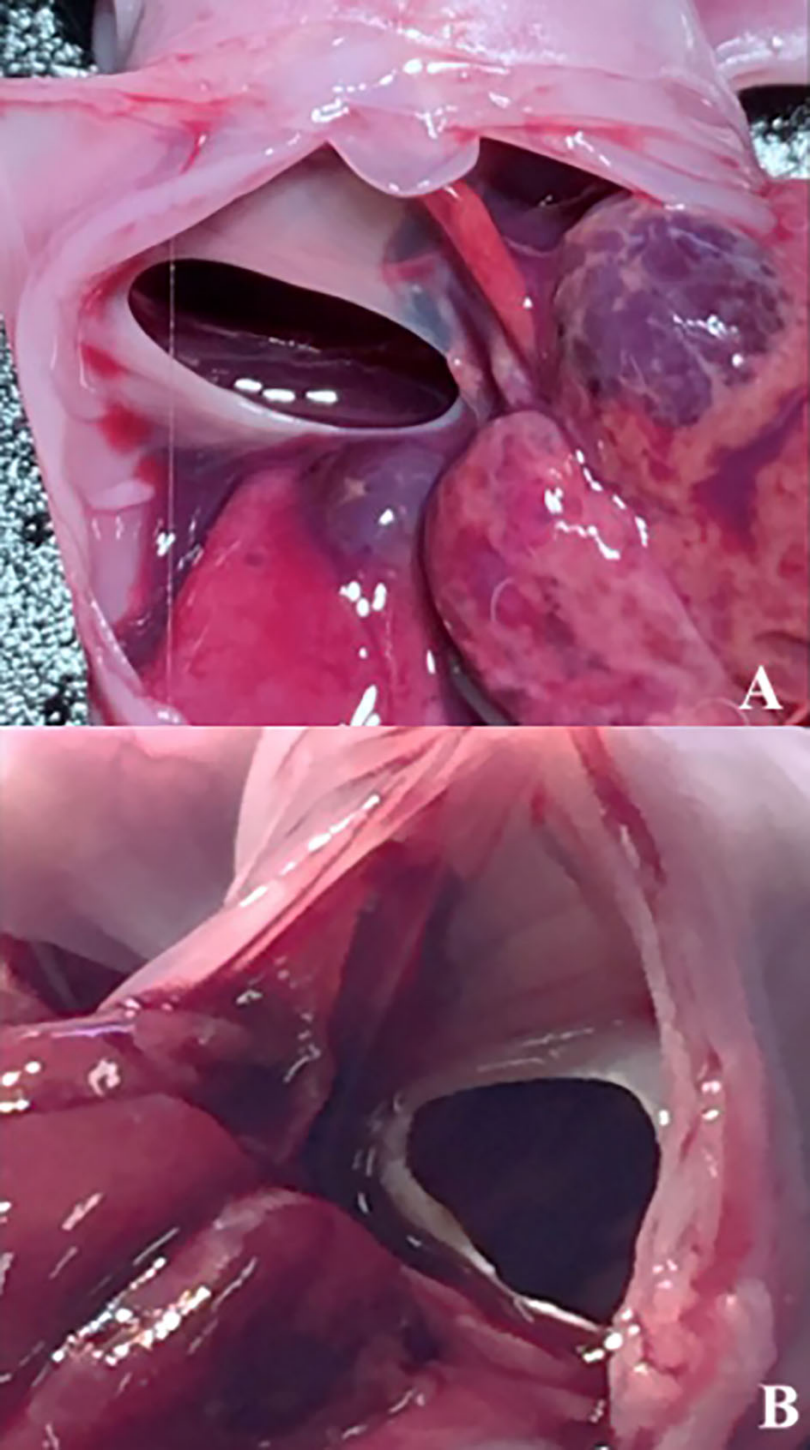
C-type size of congenital diaphragmatic hernia on the right (**A**) and left (**B**) sides of neonate rabbits.

Fetuses were harvested on the 30th day of pregnancy as described. The neonates were weighed (body weight) and an intramuscular dose of 1 mL of ketamine/xylazine was applied. The abdomen was opened to confirm the presence and size of CDH. After that, the lungs and the heart were removed and weighed (total lung weight, left lung weight, right lung weight, and heart weight), being then immersed in 10% formaldehyde for preservation (Figure 1).

Samples were then dehydrated in increasing alcohol concentrations, cleared with xylene, and embedded in paraffin. Blocks were sectioned with a microtome (Leica RM 2145, Leica, Germany) into 5-µm sections, then mounted on histological slides.

For immunohistochemistry, slides were dewaxed with xylene for 5 min and rehydrated with ethanol. The slides were incubated overnight with primary antibodies diluted in 5% bovine serum albumin: VEGFR2 (Santa Cruz Biotechnology, USA), Ki-67 (Sigma-Aldrich, USA), and lectin *Ulex europaeus* (Sigma-Aldrich).

### Histometric analysis

Five samples per group were used to perform the histometric analysis. Histological sections in the short-axis cross-section (4 μm) from the left and right ventricles were stained with hematoxylin-eosin and photos were taken with a photomicroscope (Leica DMR, Leica Microsystems Wetzlar GmbH, Switzerland). The histometric parameters measured were left ventricular wall thickness (LVWT), right ventricular wall thickness (RVWT), left ventricular area (LVA), right ventricular area (RVA). Five samples per group were measured using the imaging software NIS Elements 3.2 (Nikon Inc., USA).

### Image and data acquisition

The slides were analyzed with a microscope (Nikon H550L) and photographed (Nikon DSFi1). For each slide, ten photos were acquired: 5 of each ventricular wall, covering the entire thickness of the wall, in an epicardium-to-endocardium direction. Both the left ventricle (LV) and the right ventricle (RV) were photographed for analysis and comparison. Myocardial capillary density was manually determined. All vessels in each of the five slices were counted, and a ratio of the number of vessels/field area was determined. Ki-67 and VEGFR2 expressions were determined using a color threshold method (ImageJ, National Institutes of Health, USA). The area marked for Ki67 and VEGFR2 was determined as a ratio of total area.

### Statistical analysis

Data analysis was performed using GraphPad 8.0 (GraphPad Prism Software, USA). We performed a non-parametric one-way ANOVA and Kruskal-Wallis test, with Dunn's post-test when appropriate. Significance was considered when P<0.05.

## Results

**Figure 2:**
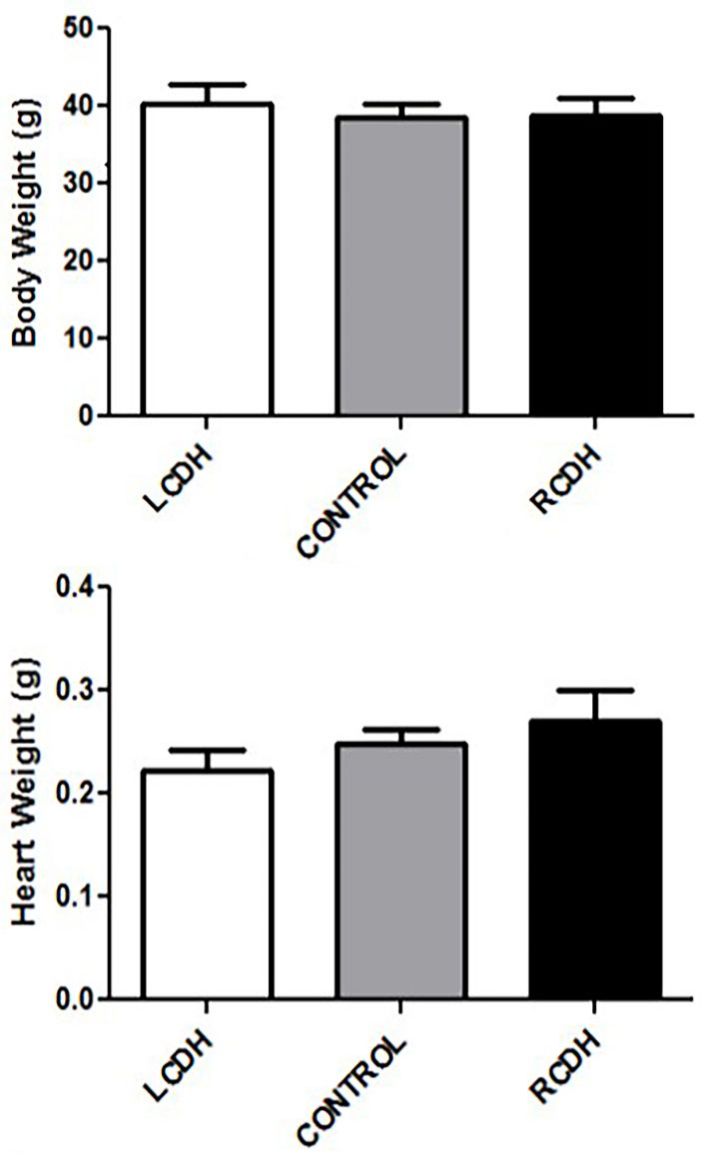
Morphometric comparison of Control, left-sided congenital diaphragmatic hernia (LCDH), and right-sided congenital diaphragmatic hernia (RCDH) groups. Data are reported as means and SD (P>0.05, ANOVA)

There was no significant difference in total body weight (P=0.702) or heart weight (P=0.165). Total lung weight (TLW): There was a significant difference among the groups where the Control was heavier than the LCDH and RCDH groups (P<0.05). There was no difference between LCDH and RCDH to TLW. Left lung weight (LLW): There was a significant difference among the groups where the Control was heavier than LCDH (P<0.05) but not than RCDH (P>0.05). There was no difference between LCDH and RCDH to LLW. Right lung weight (RLW): There was a significant difference among the groups where the Control was heavier than LCDH and RCDH (P<0.05). There was no difference between LCDH and RCDH to RLW (Figure 2).

### Histometric analysis

There were significant statistical differences among LWT, RWT, and LVA, where the LCDH group was decreased, compared with the Control and RCDH groups (P<0.05).

### Immunohistochemistry

**Figure 3:**
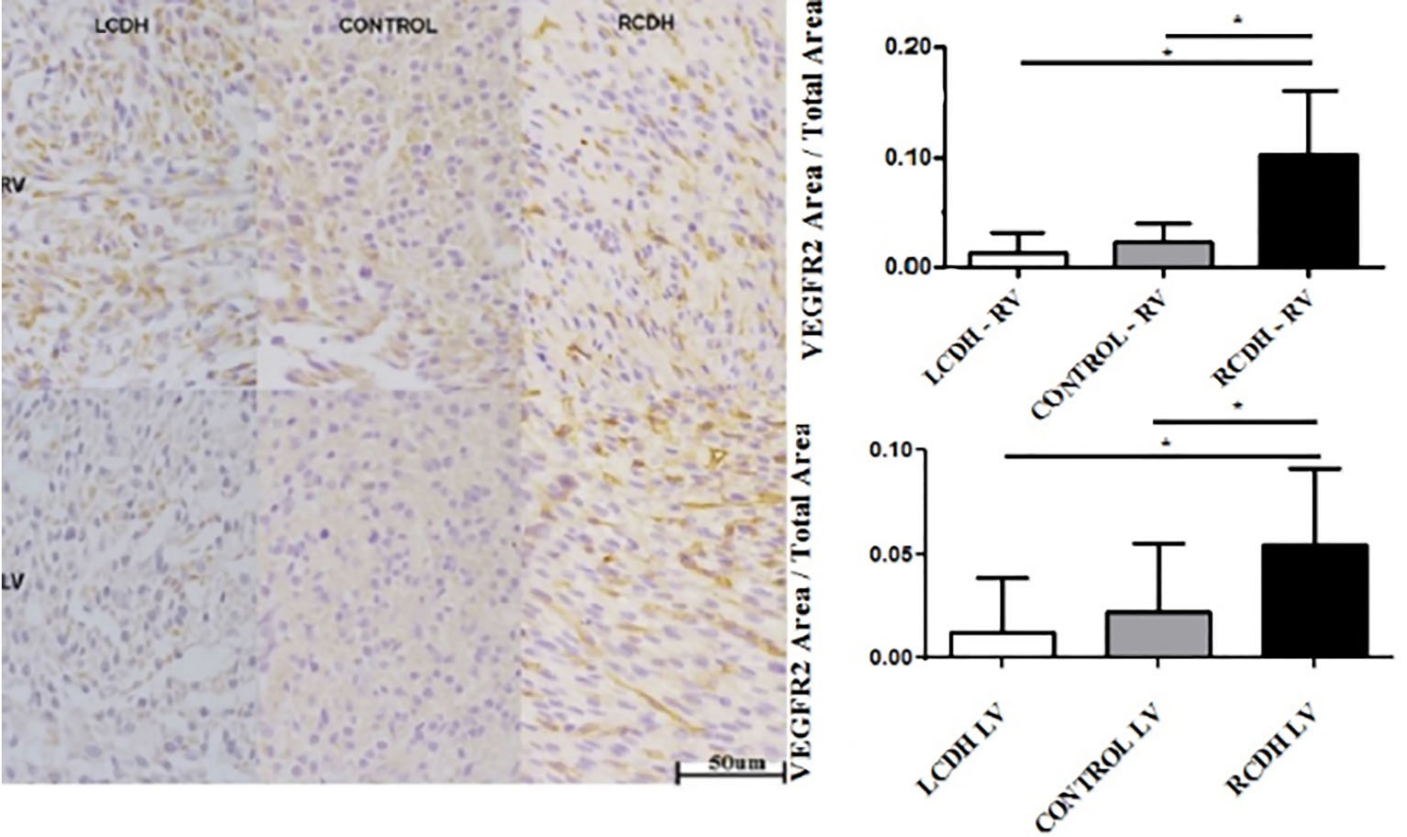
Photomicrographs (scale bar 50 μm) and graphs of VEGFR2 expression comparison in the right (RV) and left (LV) ventricles in Control, left-sided congenital diaphragmatic hernia (LCDH), and right-sided congenital diaphragmatic hernia (RCDH) groups. Data are reported as means and SD. *P<0.05 (ANOVA).

VEGFR2 expression increased significantly in both ventricles of the RCDH group compared to the Control and LCDH groups (P<0.0001 for both ventricles) (Figure 3).

**Figure 4:**
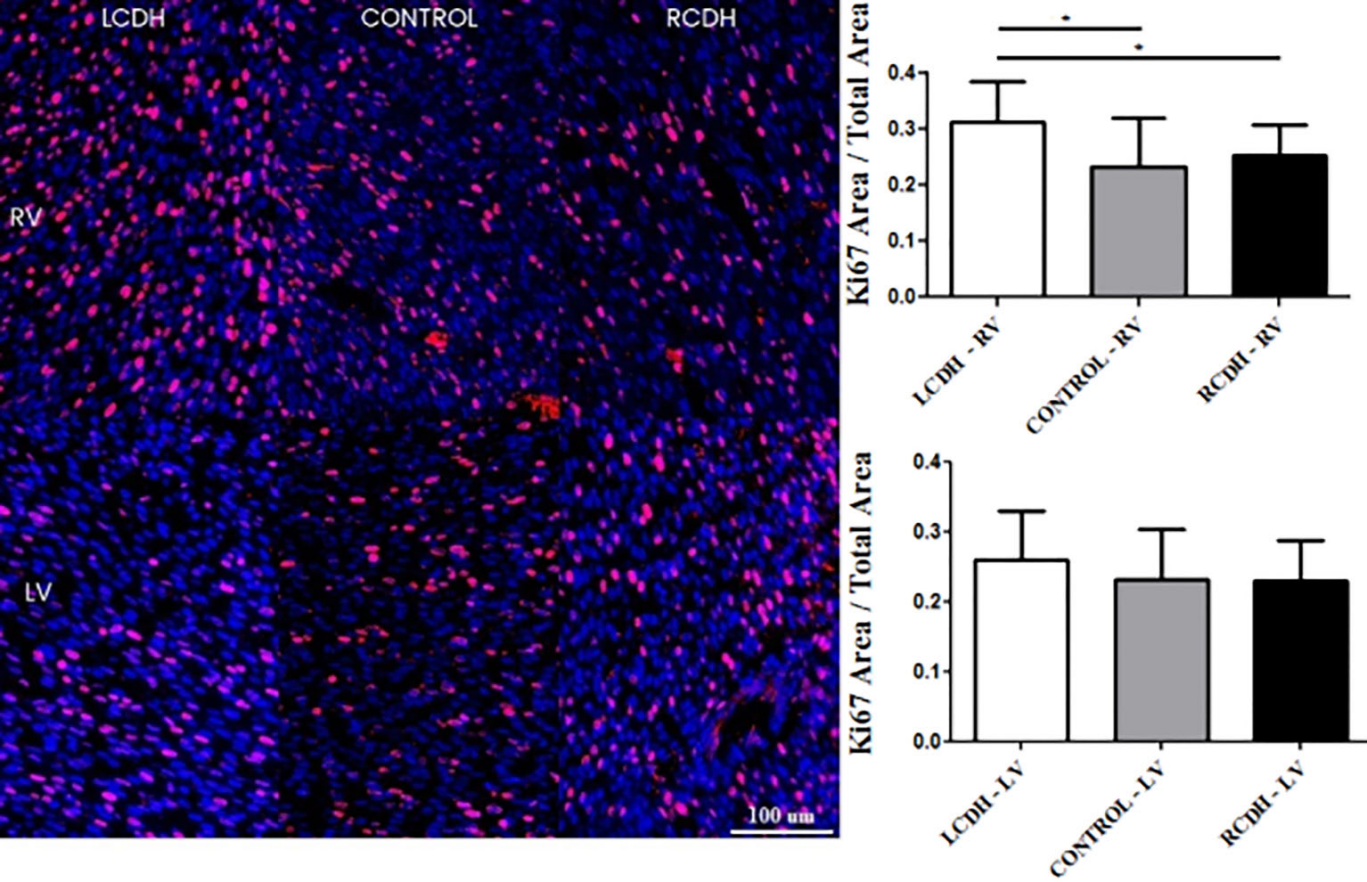
Photomicrographs (scale bar 100 μm) and graphs of Ki67 expression comparison in the right (RV) and left (LV) ventricles of the Control, left-sided congenital diaphragmatic hernia (LCDH), and right-sided congenital diaphragmatic hernia (RCDH) groups. Data are reported as means and SD. *P<0.05 (ANOVA).

Ki-67 expression was increased in the right ventricle of the LCDH group (P<0.0001). There was no difference in Ki-67 among groups in the left ventricle (P=0.055) (Figure 4).

### Capillary density measured by lectin

**Figure 5:**
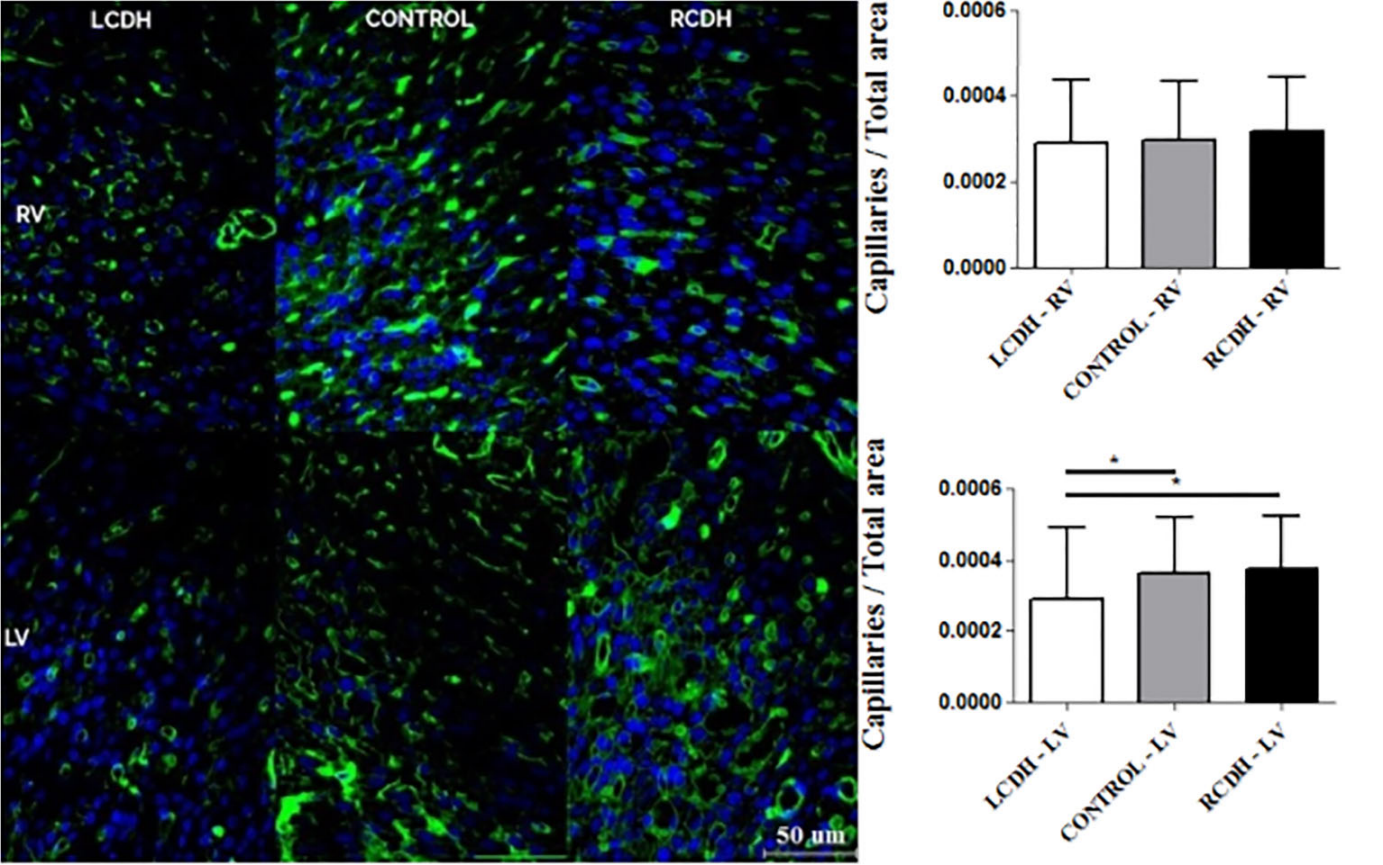
Photomicrographs (scale bar 50 μm) and graphs of lectin immunostaining comparison in the right (RV) and left (LV) ventricles of the Control, left-sided congenital diaphragmatic hernia (LCDH), and right-sided congenital diaphragmatic hernia (RCDH) groups. Data are reported as means and SD. *P<0.05 (ANOVA).

Capillary density was significantly reduced in the left ventricle of the LCDH group (P=0.002). There was no difference in capillary density among groups in the right ventricle (P=0.321). We also divided the lectin by the LVWT and RVWT and did not observe a difference between the capillarity density by thickness (Figure 5).

## Discussion

Cardiac adaptations in CDH have been previously studied both in clinical and experimental settings. The hypothesis is that the cardiorespiratory pathophysiology in CDH (pulmonary arterial hypertension) leads to increased right ventricular strain, especially after birth, with the transition to post-fetal circulation. Right ventricular function in CDH seems to go through adaptation mechanisms like other causes of pulmonary arterial hypertension, with increased right ventricular mass secondary to hypertrophy and functionally impaired early diastolic myocardial relaxation (12-14).

Moreover, although not directly affected by pulmonary arterial hypertension, the left ventricle also seems to be compromised by CDH. In the first place, reduced left ventricular mass has been described both by necroscopic analysis and echocardiography. Byrne et al. (15) described reduced left heart size and correlated the degree of heart hypoplasia with CDH severity according to the lung-to-head ratio (LHR). Tannuri (7), as previously mentioned, described reduced left ventricular wall thickness in the rabbit model used in our work. Functionally, there is also evidence for left ventricular impairment, both systolic and diastolic, leading to increased right ventricular strain through increased pulmonary venous resistance (15,16). Experimentally, Manso et al. (17) conducted echocardiography studies of fetal rabbits with surgically induced CDH but did not find a significant difference in left ventricular ejection fraction (17).

Considering the extensive evidence for cardiac implications of CDH, we studied the expression of three essential elements involved in the development and function of the myocardium. Pelizzo et al. (8) first identified differences in the number of intramyocardial arterioles in a *postmortem* study of seven hearts of fetuses with severe CDH. In the study, a higher total density was noted in the CDH group compared with Controls, although there was a reduction in the number of vessels in the left ventricle section in the CDH group. Our results are partially similar to theirs regarding the left ventricle, highlighting the role of cardiac microenvironment depending on the different cytokines/mediator profiles.

To our knowledge, this is the first study to evaluate the expression of VEGFR2, lectin, and Ki67 in the heart of rabbit fetuses with CDH. Although the immunostaining of Ki67 is predominant in the myocardium, it can also indicate proliferative activity in some cardiac vessels. The increase of Ki67 in the right ventricle could represent an essential proliferative activity of myocardium cells. In a previous study, we evaluated the histological aspects of the myocardium by measuring the smallest myocyte diameter, the largest diameter of the nucleus, the smallest diameter of the nucleus, and the mitotic myocyte count. We found an increased mitotic myocyte count in the CDH rabbit group (P<0.05) (17). It seems that the right ventricle begins its adaptation before birth, either by the mechanical effect of compression or by future pulmonary vascular resistance.

In this study, the results were somewhat surprising. The findings indicated that, first, the left and right ventricles responded differently to the effects of the abdominal content herniation. Second, left- and right-sided hernias induced different types of myocardial response. The increase in the expression of VEGFR2 caused by RCDH on both ventricles (P<0.0001) would, at first, be associated with changes in the capillary density of the myocardium, with more or less angiogenesis and vessel density per area. According to our results, the RCDH group had no difference in capillary density (P=0.321) compared to the Control group.

VEGFR-2 signaling produces several cellular responses, including intense mitogenic and survival signals for endothelial cells and their precursors (18); thus, the silencing of VEGFR-2 produces reduction or abolition of vasculogenesis in rats (19). On the other hand, VEGFR-1 acts on the development of blood vessels, negatively modulating the division of endothelial cells, as it inhibits signaling by VEGFR-2 (20). Mice with gene silencing for VEGFR-1 do not survive until the end of pregnancy and show hypertrophy and disorganization of the vascular bed (21). Lower capillary density was observed in the left ventricle in the LCDH group (P=0.002), but this finding was absent in the RCDH group. One possibility is that anatomical factors determine different effects on the left and right ventricles. Due to its position, the heart would be compressed against the left thoracic cavity when compression is on the right. On the other hand, when compression is on the left, the heart is compressed against the right lung, a more complacent medium, which could lead to less damage to the myocardium. This could explain the significant increase in VEGFR2 seen in the RCDH group. The left herniation (mostly when the liver is up) allows a more direct compression on the left side of the heart that could explain the hypoplasia of the left cardiac ventricle that we commonly found in the LCDH.

Neonates with CDH have LV diastolic dysfunction resulting from a combination of LV hypoplasia and difficult filling due to septal arching that increases RV afterload. Patel et al. (22), in a multicenter registry study, found that 55% of patients with RV dysfunction also had LV dysfunction. LV dysfunction commonly occurs in the RV dysfunction setting and is associated with outcomes in infants with CDH (23,24).

Our study had some limitations. First, the rabbit surgical CDH model studies the lungs and heart after an artificial defect is created near the end of the rabbit's pregnancy. Unlike clinical situations in which the diaphragm defect is present since embryogenesis, the surgical model leads to a shorter duration of herniation and competition for fetal chest space compared with CDH in humans. Second, due to the delicate nature of the surgical manipulation of the fetal diaphragm, herniation outcomes may vary. On the one hand, this variability reflects well the different severity of defects seen clinical practice. On the other hand, it may be a confounding factor when analyzing the impact of herniation. In our study, we used only type C and D defects to standardize the type of defect and reduce bias from animals with less severe defects.

With its dynamic, beating physiology, the heart poses a significant challenge for the investigation of mechanical factors. Fox et al. (25) described a compression model using a microdevice that applies constant pressure to *ex vivo* lung tissue. This could represent a new possibility to better isolate and study the effects of mechanical compression on the heart in CDH models and to better understand its implications.

The relationship between fetal cardiac hypoplasia and clinical outcome remains, so far, unresolved (26). An increase in outflow fraction and LV size change in the heart and consequently in postoperative recovery and clinical management due to the dispersion of myocardial function in CDH (16). In a case-control study, echocardiographic data showed that infants with CDH have LV and RV dysfunction on the first day of life but without an increase in pulmonary artery pressure. The LV dysfunction could be of primary origin due to its smaller size or could be secondary to the RV dysfunction. These dysfunctions imply management with vasoactive drugs to control pulmonary arterial hypertension and may be targets of new therapeutic strategies (16). This decrease in LV size is associated with worse pulmonary hypertension with higher mortality (27), in addition to a longer hospital stay (28) and greater use of extracorporeal membrane oxygenation and inhaled nitric oxide (29).

Our experiments confirmed the identification of myocardial alterations with the identification of vascular reduction of the LV, which may justify the clinical prognosis and, therefore, the need for echocardiographic monitoring of LV dimensions and pulmonary pressure in the follow-up of CDH.

In conclusion, our study showed that different patterns of capillary proliferation, activation, and density in the myocardium of the ventricles of newborn rabbits were related to the cardiac microenvironment and that left and right ventricles responded differently to CDH-associated compression in our surgical model of CDH.

## References

[1] Chandrasekharan PK, Rawat M, Madappa R, Rothstein DH, Lakshminrusimha S (2017). Congenital diaphragmatic hernia - a review. Matern Health Neonatol Perinatol.

[2] Brownlee EM, Howatson AG, Davis CF, Sabharwal AJ (2009). The hidden mortality of congenital diaphragmatic hernia: a
20-year review. J Pediatr Surg.

[3] Keijzer R, Liu J, Deimling J, Tibboel D, Post M (2000). Dual-hit hypothesis explains pulmonary hypoplasia in the nitrofen
model of congenital diaphragmatic hernia. Am J Pathol.

[4] George DK, Cooney TP, Chiu BK, Thurlbeck WM (1987). Hypoplasia and immaturity of the terminal lung unit (acinus) in
congenital diaphragmatic hernia. Am Rev Respir Dis.

[5] Siebert JR, Haas JE, Beckwith JB (1984). Left ventricular hypoplasia in congenital diaphragmatic
hernia. J Pediatr Surg.

[6] Schwartz SM, Vermilion RP, Hirschl RB (1994). Evaluation of left ventricular mass in children with left-sided
congenital diaphragmatic hernia. J Pediatr.

[7] Tannuri U (2001). Heart hypoplasia in an animal model of congenital diaphragmatic
hernia. Rev Hosp Clin Fac Med Sao Paulo.

[8] Pelizzo G, Bussani R, Zandonè L, Custrin A, Bellieni CV, De Silvestri A (2016). Cardiac adaptation to severe congenital diaphragmatic
hernia. Fetal Pediatr Pathol.

[9] Sbragia L, Nassr AC, Gonçalves FL, Schmidt AF, Zuliani CC, Garcia PV (2014). VEGF receptor expression decreases during lung development in
congenital diaphragmatic hernia induced by nitrofen. Braz J Med Biol Res.

[10] Fauza DO, Tannuri U, Ayoub AA, Capelozzi VL, Saldiva PH, Maksoud JG (1994). Surgically produced congenital diaphragmatic hernia in fetal
rabbits. J Pediatr Surg.

[11] Pringle KC (1986). Human fetal lung development and related animal
models. Clin Obstet Gynecol.

[12] Patel N, Mills JF, Cheung MMH (2009). Assessment of right ventricular function using tissue Doppler
imaging in infants with pulmonary hypertension. Neonatology.

[B13] Patel N, Massolo AC, Kipfmueller F (2020). Congenital diaphragmatic hernia-associated cardiac
dysfunction. Semin Perinatol.

[14] Chin KM, Kim NH, Rubin LJ (2005). The right ventricle in pulmonary hypertension. Coron Artery Dis.

[15] Byrne FA, Keller RL, Meadows J, Miniati D, Brook MM, Silverman NH (2015). Severe left diaphragmatic hernia limits size of fetal left heart
more than does right diaphragmatic hernia. Ultrasound Obstet Gynecol.

[16] Massolo AC, Paria A, Hunter L, Finlay E, Davis CF, Patel N (2019). Ventricular dysfunction, interdependence, and mechanical
dispersion in newborn infants with congenital diaphragmatic
hernia. Neonatology.

[17] Manso PH, Figueira RL, Prado CM, Gonçalves FL, Simões AL, Ramos SG (2015). Early neonatal echocardiographic findings in an experimental
rabbit model of congenital diaphragmatic hernia. Braz J Med Biol Res.

[18] Bernatchez PN, Soker S, Sirois MG (1999). Vascular endothelial growth factor effect on endothelial cell
proliferation, migration, and platelet-activating factor synthesis is
Flk-1-dependent. J Biol Chem.

[19] Shalaby F, Ho J, Stanford WL, Fischer KD, Schuh AC, Schwartz L (1997). A requirement for Flk1 in primitive and definitive hematopoiesis
and vasculogenesis. Cell.

[20] Roberts DM, Kearney JB, Johnson JH, Rosenberg MP, Kumar R, Bautch VL (2004). The vascular endothelial growth factor (VEGF) receptor Flt-1
(VEGFR-1) modulates Flk-1 (VEGFR-2) signaling during blood vessel
formation. Am J Pathol.

[21] Fong GH, Rossant J, Gertsenstein M, Breitman ML (1995). Role of the Flt-1 receptor tyrosine kinase in regulating the
assembly of vascular endothelium. Nature.

[22] Patel N, Lally PA, Kipfmueller F, Massolo AC, Luco M, Van Meurs KP (2019). Ventricular dysfunction is a critical determinant of mortality in
congenital diaphragmatic hernia. Am J Respir Crit Care Med.

[23] Dao DT, Patel N, Harting MT, Lally KP, Lally PA, Buchmiller TL (2020). Early left ventricular dysfunction and severe pulmonary
hypertension predict adverse outcomes in “low-risk” congenital diaphragmatic
hernia. Pediatr Crit Care Med.

[24] Critser PJ, Levy PT (2020). Risk assessment and monitoring of right ventricular function in
congenital diaphragmatic hernia. Ann Am Thorac Soc.

[25] Fox ZD, Jiang G, Ho KKY, Walker KA, Liu AP, Kunisaki SM (2018). Fetal lung transcriptome patterns in an *ex vivo*
compression model of diaphragmatic hernia. J Surg Res.

[26] Patel N, Massolo AC, Kraemer US, Kipfmueller F (2022). The heart in congenital diaphragmatic hernia: Knowns, unknowns,
and future priorities. Front Pediatr.

[27] Karpuz D, Giray D, Celik Y, Hallioglu O (2018). Prognostic markers in congenital diaphragmatic hernia: left
ventricular diameter and pulmonary hypertension. Pediatr Int.

[28] Coffman ZJ, McGahren ED, Vergales BD, Saunders CH, Vergales JE (2019). The effect of congenital diaphragmatic hernia on the development
of left-sided heart structures. Cardiol Young.

[29] Kailin JA, Dhillon GS, Maskatia SA, Cass DL, Shamshirsaz AA, Mehollin-Ray AR (2017). Fetal left-sided cardiac structural dimensions in left-sided
congenital diaphragmatic hernia - association with severity and impact on
postnatal outcomes. Prenat Diagn.

